# Investigation of the Effectiveness of Silicon Nitride as a Reinforcement Agent for Polyethylene Terephthalate Glycol in Material Extrusion 3D Printing

**DOI:** 10.3390/polym16081043

**Published:** 2024-04-10

**Authors:** Nikolaos Michailidis, Markos Petousis, Vassilis Saltas, Vassilis Papadakis, Mariza Spiridaki, Nikolaos Mountakis, Apostolos Argyros, John Valsamos, Nektarios K. Nasikas, Nectarios Vidakis

**Affiliations:** 1 Physical Metallurgy Laboratory, Mechanical Engineering Department, School of Engineering, Aristotle University of Thessaloniki, 54124 Thessaloniki, Greece; nmichail@auth.gr (N.M.); aargyros@auth.gr (A.A.); 2 Centre for Research & Development of Advanced Materials (CERDAM), Centre for Interdisciplinary Research and Innovation, Balkan Centre, Building B’, 10th Km Thessaloniki-Thermi Road, 57001 Thessaloniki, Greece; 3 Department of Mechanical Engineering, Hellenic Mediterranean University, 71410 Heraklion, Greece; markospetousis@hmu.gr (M.P.); mspyridaki@hmu.gr (M.S.); mountakis@hmu.gr (N.M.); valsamos@hmu.gr (J.V.); 4 Department of Electronic Engineering, Hellenic Mediterranean University, 73133 Chania, Greece; saltas@hmu.gr; 5 Institute of Electronic Structure and Laser of the Foundation for Research and Technology-Hellas (IESL-FORTH)—Hellas, N. Plastira 100m, 70013 Heraklion, Greece; v.papadakis@uniwa.gr; 6 Department of Industrial Design and Production Engineering, University of West Attica, 12243 Athens, Greece; 7 Division of Mathematics and Engineering Sciences, Department of Military Sciences, Hellenic Army Academy, 16673 Vari, Greece; nasikas@sse.gr

**Keywords:** polyethylene terephthalate glycol (PETG), silicon nitride (Si_3_N_4_), nanocomposites, material extrusion (MEX), three-dimensional (3D) printing, mechanical performance, broadband dielectric spectroscopy (BDS)

## Abstract

Polyethylene terephthalate glycol (PETG) and silicon nitride (Si_3_N_4_) were combined to create five composite materials with Si_3_N_4_ loadings ranging from 2.0 wt.% to 10.0 wt.%. The goal was to improve the mechanical properties of PETG in material extrusion (MEX) additive manufacturing (AM) and assess the effectiveness of Si_3_N_4_ as a reinforcing agent for this particular polymer. The process began with the production of filaments, which were subsequently fed into a 3D printer to create various specimens. The specimens were manufactured according to international standards to ensure their suitability for various tests. The thermal, rheological, mechanical, electrical, and morphological properties of the prepared samples were evaluated. The mechanical performance investigations performed included tensile, flexural, Charpy impact, and microhardness tests. Scanning electron microscopy and energy-dispersive X-ray spectroscopy mapping were performed to investigate the structures and morphologies of the samples, respectively. Among all the composites tested, the PETG/6.0 wt.% Si_3_N_4_ showed the greatest improvement in mechanical properties (with a 24.5% increase in tensile strength compared to unfilled PETG polymer), indicating its potential for use in MEX 3D printing when enhanced mechanical performance is required from the PETG polymer.

## 1. Introduction

Additive manufacturing (AM) technology has been the subject of intense research and development for over 30 years, resulting in constant progress and innovative techniques that constitute a true revolution in manufacturing. AM processes include most notably stereolithography (SLA) [1], fused deposition modeling (FDM) [2], three-dimensional printing (3DP) [3], selective laser sintering (SLS) [4], laminated objective manufacturing (LOM) [5], and laser metal deposition (LMD) [6]. AM provides the opportunity to create parts with complex geometries in a shorter period of time and at a significantly lower cost [7] than traditional manufacturing techniques [8]. AM has important and innovative applications in a variety of fields, such as engineering [9], aerospace [10,11,12,13,14,15], the automotive industry [11,12,16,17], energy [18], the use of ceramic materials [19], electronics [20,21], defense [22,23], and medicine [24,25,26,27,28,29].

Fused filament fabrication (FFF) or material extrusion (MEX) can easily be utilized for the processing of polymers and reinforced polymers because it is a simple procedure that involves feeding an extrusion-suitable thermoplastic filament into a nozzle and fabricating the desired 3D sample [30,31]. It should be mentioned that some settings must be properly and carefully set during each FFF procedure because they can influence the mechanical performance of the 3D-printed sample [32,33]. Those could be, among others, the printing speed, the layer thickness, the temperature of the nozzle, the bed, and the chamber, etc. [34,35].

Polyethylene terephthalate glycol (PETG) is the most common thermoplastic polymer resin among polyesters. It is a modified polyethylene terephthalate (PET) polymer filled with glycol that provides better processability in the FFF technique [36,37]. It possesses some advanced properties compared to PET, such as chemical alkali resistance, high shrinkage, transparency, gloss, low haze, good printability [38], and processability, along with the ability to be easily mixed with blends [39]. It can be useful in applications that require more enhanced mechanical properties than PET; moreover, its 3D-printing FFF process is easy to use, and it can be utilized for food packaging [38], engineering [40], and medical applications [41], among others.

Many industries tend to choose ceramics for specific applications owing to their advanced properties [42] such as high hardness and high melting temperature (Tm). Such quantities are utilized in applications featuring high loads, such as in cutting tools [43,44]. Silicon nitride (Si_3_N_4_) is a ceramic material that exhibits extraordinary properties, such as high durability, potency, crack-withstanding ability, and resistance to sudden temperature changes [45,46,47]. Therefore, such ceramics and analogous materials are suitable for specific applications that require optimal operation under harsh working conditions, such as bearings [48], hard coatings, cutting tools [49], spark plugs [50,51], and engine parts that operate at high temperatures [52]. According to many studies, the presence of nitride nanoparticles can be beneficial to the characteristics and behavior of polymer nanocomposites with regard to their thermomechanical properties [47,53,54]. In addition to its excellent mechanical properties as a ceramic material, Si_3_N_4_ is considered a bioceramic that can be useful for medical or healthcare applications. It is a subject of interest in the field of biomedicine because of its biocompatibility and superior chemical, physical, and mechanical properties, which are necessary for applications such as implants [55,56] and scaffolds [57,58]. Si_3_N_4_ ceramics are also characterized by their bacteriostatic properties, which can be useful in a variety of biomedical applications [45,46]. Recently, they have been used in biomedical applications such as in 3D printing using material extrusion (MEX) [59,60] and vat photopolymerization (VPP) [61,62] methods.

In this research endeavor, various composites were developed by blending PETG as the matrix material with Si_3_N_4_ ceramic as the filler, with the aim of evaluating their performance in a range of tests. This study examined the impact of Si_3_N_4_ nanoparticles as reinforcing agents for PETG thermoplastic. The objective was to enhance the mechanical properties of PETG thermoplastic in MEX 3D printing, with an emphasis on developing a process that could be scaled up for industrial production. This enhancement is intended to improve the durability of components, particularly in applications where PETG thermoplastic is the preferred polymer, thus potentially expanding its range of use. It is worth noting that this investigation into PETG/Si_3_N_4_ nanocomposites, especially in terms of their mechanical properties, is novel in the literature, particularly within the realm of additive manufacturing (AM) or MEX 3D printing technologies.

In this study, PETG was used in conjunction with Si_3_N_4_ to create composites with five filler percentages (2.0, 4.0, 6.0, 8.0, and 10.0 wt.%). The corresponding mixtures were created and used to fabricate filaments via material extrusion (MEX). The produced filaments were later fed into a three-dimensional printer (3D-P) to fabricate the desired specimens in accordance with the respective international standards, for which the specimens subsequently underwent a variety of tests. The same settings were applied for the fabrication of all the samples so that the results would be comparable. The samples were subjected to a series of mechanical tests, including tensile, flexural, impact, and microhardness tests. Thermogravimetric analysis (TGA), differential scanning calorimetry (DSC), and Raman spectroscopy were used to determine the thermal properties of the composites. Their electrical/dielectric properties over a broad frequency range were examined, and scanning electron microscopy (SEM) and energy-dispersive X-ray spectroscopy (EDS) were employed to reveal the structural characteristics and chemical compositions of the samples. Micro-CT (μ-CT) was used to determine the dimensional accuracy and internal structure of the fabricated samples. A significant enhancement in the mechanical properties was exhibited by the 6.0 wt.% composite made of Si_3_N_4,_ which led to this specific filler percentage composite being considered suitable for providing the nanocomposites with reinforced mechanical properties. However, the electrical/dielectric response of PETG/Si_3_N_4_ composites is mainly dependent on the electrical behavior of the polymer matrix. This study aims to achieve the following:

- Examine the ability of Si_3_N_4_ to enhance the mechanical properties and reinforce the performance of the PETG polymer.
- Determine the contribution of Si_3_N_4_ to the electrical/dielectric properties of the PETG polymer.
- Investigate and provide evidence for the suitability of PETG/Si_3_N_4_ composites for use as 3D printable materials for the fabrication of various parts that can serve a constantly increasing variety of applications in demanding and extreme environments.

## 2. Materials and Methods

**Figure 1:**
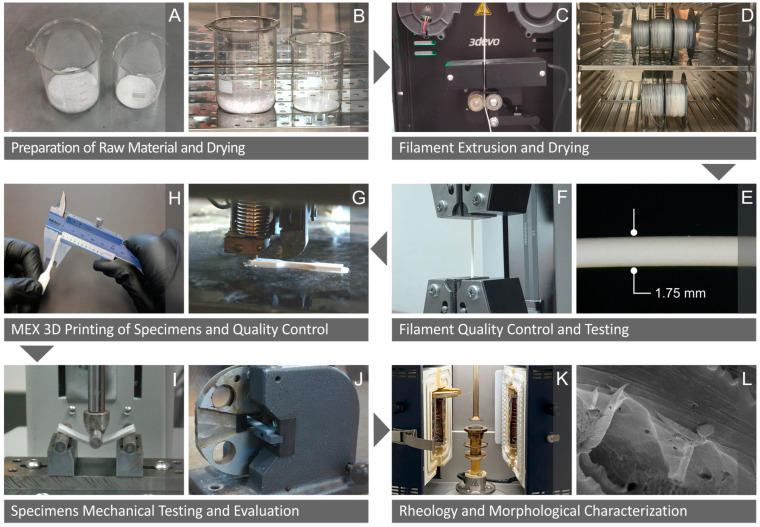
The steps followed in the present study include (**A**) the preparation of the unprocessed materials and (**B**) their drying; (**C**) the production of the filaments through extrusion and (**D**) their subsequent drying; (**E**) inspection of quality and (**F**) mechanical testing; the (**G**) material extrusion of the three-dimensional specimens and (**H**) the inspection of their quality; (**I**,**J**) examination and evaluation of mechanical properties; and (**K**) rheological and (**L**) morphological investigation.

The present work involved a series of steps, summarized in Figure 1, regarding the preparation of the raw materials, filaments, and specimens as well as the tests conducted on them. Figure 1A,B display the raw materials prepared and dried in an oven to remove any absorbed moisture. As shown in Figure 1C,D, the filaments were extruded and allowed to dry in an oven, while Figure 1E,F show their quality inspection and mechanical testing. Figure 1G,H show the material extrusion 3D-P of the corresponding specimens and their quality control. Figure 1I,J concern the mechanical testing and evaluation of the specimens, while their rheological and morphological characteristics are shown in Figure 1K,L.

### 2.1. Materials

Polyethylene Terephthalate Glycol (PETG) was supplied by Felfil Srl (Torino, Italy) in pellet form. Nanographi (Ankara, Turkey) was used as the source of silicon nitride (Si_3_N_4_) nanoparticles (Ankara, Turkey).

### 2.2. Preparation of the PETG/Si_3_N_4_ Filament and 3D Printing

Preparation of PETG/x wt.% Si_3_N_4_ filaments began by measuring the correct quantities of both materials and then using them to synthesize the desired compounds. As to the definition of the filler percentage limit, it was set according to the results of preliminary tests conducted on the samples, while gradually increasing the filler quantity. Increasing the amount of additive was discontinued when the performance of the samples ceased to provide useful results and information. Apart from the pure PETG quantity, which was prepared, five different mixtures of PETG/*x* wt.% Si_3_N_4_ were also compounded, namely, PETG/2.0 wt.% Si_3_N_4_, PETG/4.0 wt.% Si_3_N_4_, PETG/6.0 wt.% Si_3_N_4_, PETG/8.0 wt.% Si_3_N_4_, and PETG/10.0 wt.% Si_3_N_4_. After being left in the oven overnight at 80 °C to dry, the filaments were fed onto a 3D Evo Composer single-screw 450 extruder supplied by 3devo (3devo B. V., Utrecht, The Netherlands). It should be noted that the diameter of the filaments was monitored during extrusion using a filament diameter sensor with the ability to make micro-adjustments, if needed, to the extrusion speed. The available literature provided this study with the necessary information about the extrusion parameters. The diameter for all filaments remained almost steady at a range between 1.65 mm and 1.85 mm, which is considered suitable for the 3D-P of the specimens. The filaments, which were dried overnight at 80 °C, were subsequently supplied to an FFF Intamsys Funmat HT 3D printer purchased from Intamsys Technology Co., Ltd. (Shanghai, China) for the production of 3D-P specimens. They were designed using 3D Autodesk^®^ Fusion 360™ software (Autodesk^®^, Inc, San Francisco, CA, USA, https://www.autodesk.com/campaigns/education/fusion-360, accessed on 20 November 2023) and exported into Standard Tesselation Language (STL) format files.

### 2.3. SEM of Si_3_N_4_ and EDS Analysis

Scanning electron microscopy (SEM) analysis was conducted by capturing fracture and side surface images of the specimens using an electron Jeol JSM-IT700HR (Jeol Ltd., Tokyo, Japan) field-emission scanning electron microscope. The chemical elements of the samples were detected via EDS analysis using the same device employed for SEM. The chemical composition of Si_3_N_4_ was investigated using the same procedure. The scanning electron microscope was operated in high-vacuum mode with a 5 kV acceleration voltage, and the samples were gold-sputtered during the SEM and EDS analyses.

**Figure 2:**
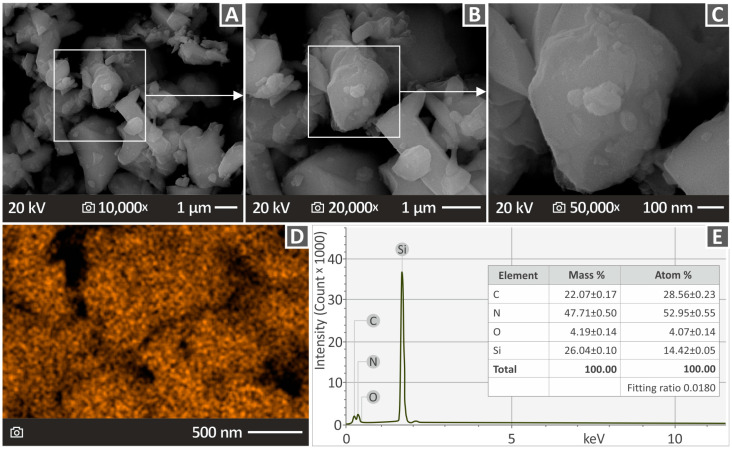
Analyzation of the unprocessed material of Si_3_N_4_ through (**A**) SEM pictures magnifying specific areas of the material at 10,000×, (**B**) 20,000×, and (**C**) 50,000× as well as (**D**) an EDS mapping image and (**E**) a chemical composition analysis via EDS.

In Figure 2A, an SEM image of Si_3_N_4_ at 10,000× magnification is presented, and the highlighted square area is shown in Figure 2B at 20,000× magnification. The highlighted square area in Figure 2B is magnified 50,000× (Figure 2C). The EDS mapping results for Si_3_N_4_ are shown in Figure 2D, and Figure 2C shows the chemical composition analysis derived from the EDS. As can be observed, high levels of Si were detected, which was an expected phenomenon.

### 2.4. Mechanical Tests

The various mechanical tests conducted on the specimens included tensile, flexural, Charpy impact, and microhardness (MH) tests. Several 3.2 mm thick V-type tensile specimens were created and tested according to the American Society for Testing and Materials (ASTM) standard D638-02a [63]. The specimens were placed between the standardized grips of an Imada MX2 (Imada Inc., Northbrook, IL, USA) tension/flexure test device in tensile mode for the tensile tests. The same machine but in flexural mode was used for flexural 3-point bending tests based on ASTM D790-10 [64] (3-point bending test with a 52.0 mm support span). ASTM D6110-04 [65] was used for impact testing, which was conducted using a Charpy impact apparatus (Terco MT 220, Terco, Sweden). ASTM E384-17 [66] was used for M–H measurements, and a Vickers apparatus, Innova Test 300 (Europe BV, Maastricht, The Netherlands), was used on specimens whose surfaces were preliminarily fully polished under a force of 100 gF and for an indentation duration of 10 s.

**Figure 3:**
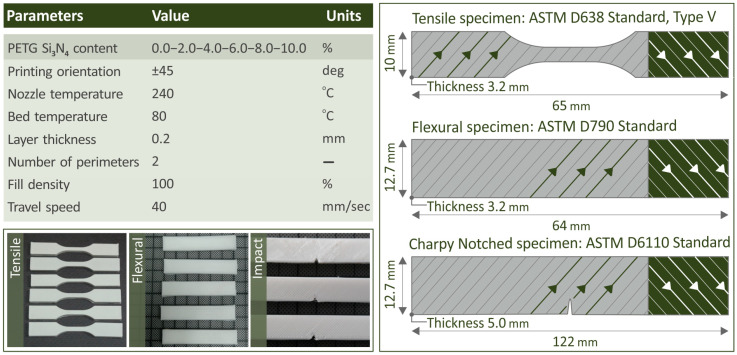
(**Top left area of the figure**) A data board including the printing parameters of the specimens, (**bottom left area of the figure**) the actual 3D-P specimens, and (**right area of the figure**) the initially designed models of the specimens. The arrows on the specimens on the right side indicate the built pattern orientation, which is changing by 90 degrees (±45 degrees) between the successive layers.

On the left side of Figure 3, the set 3D-P parameters are presented, along with some samples from the actual 3D-printed tensile, flexural, and impact specimens. The nozzle temperature was set to 240 °C, the bed temperature was 80 °C, the thickness of the layers was 0.2 mm, and the travel speed was 40 mm/s. On the right side of Figure 3, the initially designed models of the tensile, flexural, and impact specimens are presented along with their dimensions and related ASTM standards.

### 2.5. Raman Spectroscopy

A confocal LabRAM HR Raman spectrometer (HORIBA Scientific, Kyoto, Japan) was employed to obtain Raman Spectroscopy measurements under laboratory conditions. A 532 nm laser line was used for Raman signal excitation with a power of 90 mW. To help control the laser power applied to the sample, a 5% neutral-density filter was placed in the optical path of the laser beam. The laser was focused on the sample with a 50× microscope Olympus objective lens (LMPlanFL N) with a Numerical Aperture (NA) and a 10.6 mm working distance. The Raman signal was collected using the same objective lens and visualization of the sample area. The acquisition settings used in this study were as follows:

- Measurement spectral sensitivity range (50 up to 3900 cm^−1^);
- Spectral resolution (2 cm^−1^);
- Spectrometer grating (600 grooves/mm);
- Exposure time at each measurement point (10 s);
- Measurement accumulations per point (5);
- Measurement point dimensions (1.7 μm laterally and 2 μm axially).

The laser power resulting from the surface of the sample was 2 mW. 

Following each measurement, any possible discoloration or degradation caused by laser irradiation was detected by visually inspecting the irradiated areas. No such events were detected in the Raman spectroscopy measurements, as described above, ensuring that the parameters for obtaining various Raman spectra were optimal.

### 2.6. Thermogravimetric and Differential Scanning Calorimetry Analyses

The thermal behaviors of the samples derived from the PETG/x wt.% Si_3_N_4_ composites and the pure PETG were examined by means of thermogravimetric analysis (TGA) and differential scanning calorimetry (DSC). The apparatus used was a Diamond Perkin Elmer (Waltham, MA, USA) with a 40–550 °C temperature cycle at an increase rate of 10 °C/min. A Discovery Series DSC-25 DSC calorimeter (TA Instruments, New Castle, DE, USA) was used to obtain DSC measurements, along with an RSC-90 Refrigerated Cooling System. The TGA and DSC analyses were conducted in an inert environment in the presence of high-purity N_2_ (nitrogen gas).

### 2.7. Investigation of the Rheometric Behavior

A DHR-20 Discovery Hybrid Rotational Rheometer (TA Instruments, DE, USA) was used to record rheological measurements, according to ASTM D1238-13 [67], for the melt flow rate (MFR). An Environmental Test Chamber with a parallel-plate setup was used for temperature regulation, and the duration of the acquisition was set to 10 s at every measurement point, with the aim of preventing excessive heating and decomposition. Melt flow rate (MFR) and rotational rheometric tests were used to assess the flow rates of the materials at certain temperatures and previously determined pressures.

### 2.8. Broadband Dielectric Spectroscopy (BDS) Examination

Dielectric spectroscopy measurements of PETG/x wt.% Si_3_N_4_ composites were performed using an Alpha-ANB high-resolution dielectric analyzer combined with a ZGS Alpha Active sample holder and BDS 1100 module (Novocontrol Technologies GmbH & Co., Montabaur, Germany). Measurements were performed in the frequency range of 10^−2^ Hz–4 MHz at room temperature. Disk-shaped specimens with a diameter of 40 mm and a thickness of 4 mm were prepared via thermal pressing to form the dielectric material of the sandwich-structured capacitor in a two-electrode configuration. A conductive paste was applied to both sides of the specimens to ensure proper electrical contact with the gold-plated electrodes of the sample holder. The applied ac voltage was set to V_rms_ = 1 V. WinDeta software, developed by Novocontrol Technologies (Novocontrol Technologies GmbH & Co., Montabaur, Germany, https://www.novocontrol.de/php/windeta.php, accessed on 20 December 2023), was used to control the experiments and acquire data. 

The dielectric results are presented as the complex dielectric permittivity ε*(ω), dissipation factor tan(δ), and complex AC conductivity σ*(ω) according to the following equations:

(1) $$\varepsilon^{*}\left(\omega \right)=\varepsilon^{\prime }-i\varepsilon^{\prime \prime }=\frac{C\left(\omega \right)}{C_{o}}-i\frac{1}{\omega \cdot C_{o}\cdot R\left(\omega \right)}$$

(2) $$\tan\left(\delta \right)=\frac{\varepsilon^{\prime \prime }}{\varepsilon^{\prime }}$$

and

(3) $$\sigma^{*}\left(\omega \right)=\sigma^{\prime }-i\sigma^{\prime \prime }=i\omega \varepsilon_{ο}\left(\varepsilon^{*}\left(\omega \right)-1\right)=\omega \varepsilon_{ο}\varepsilon^{\prime \prime }-i\omega \varepsilon_{ο}\left(\varepsilon^{\prime }-1\right)$$

where R(ω) and C(ω) are the resistance and capacitance of the equivalent circuit in parallel connection measured using the dielectric analyzer, $C_{o}=\varepsilon_{ο}\cdot \pi \cdot r^{2}/d$ is the capacitance of the empty cylindrical sample cell with radius r and distance d between the electrodes, ω=2πf is the angular frequency, and $\varepsilon_{ο}$ is the permittivity of the vacuum. The real part of the dielectric permittivity (ε′) measures the energy storage under the effect of an applied electric field, whereas the dissipation factor tan(δ) $\varepsilon^{\prime \prime }$ is related to the energy loss within the material. 

### 2.9. Micro-Computed Tomography

The porosity and dimensional deviation of the manufactured 3D-P specimens were examined using micro-CT (μ-CT). A CT scanner, namely, a Tomoscope HV Compact 225 kV Micro Focus (Werth Messtechnik GmbH, Giessen, Germany), with a 1024 × 1024-pixel sensor was employed along with VG Studio MAX 2.2 software (Volume Graphics GmbH, Heidelberg, Germany) for data processing. Through these examinations, the effects of additives on the 3D-P structure were evaluated. A 75 L setup (72.58 μm resolution on the *X*-axis and 72.65 μm resolution on the *Y*-axis) was used to examine dimensional accuracy. A 16 L setup (15.46 μm resolution on the *X*-axis and 15.49 μm resolution on the *Y*-axis) was used to examine porosity. A total of 1600 sections per revolution were acquired for both cases.

## 3. Results

### 3.1. Raman Results for PETG/x wt.% Si_3_N_4_ Composites

**Figure 4:**
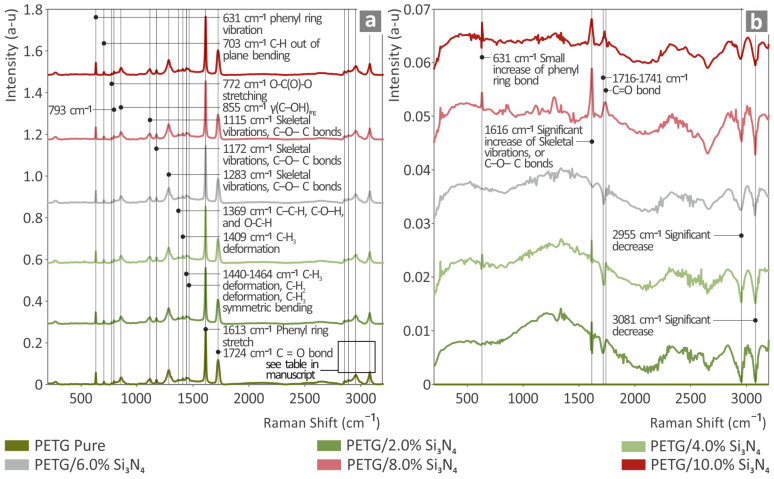
Representation of (**a**) the Raman graphs of the neat PETG and the PETG/*x* wt.% Si_3_N_4_ (*x* = 2.0, 4.0, 6.0, 8.0, and 10.0 wt.%) samples, accompanied by the (**b**) graphs created by the subtraction of the pure PETG spectral signature.

Figure 4a shows the Raman spectra of the pure PETG and PETG/x wt.% Si_3_N_4_ composites, while in Figure 4b, the results obtained by subtracting pure PETG from all the spectra of Figure 4a are presented.

In Table 1, the related Raman peaks from the pure PETG sample are presented as extracted from the literature, along with their references in brackets, indicating the work(s) from whence the data originated.

As shown in Figure 4a, as the concentration of Si_3_N_4_ increased in the PETG composite, the related Raman lines of the pure PETG differentiated in intensity. Furthermore, broad photoluminescence appeared to increase as the Si_3_N_4_ concentration increased in the spectral region from 800 to 1800 cm^−1^.

The addition of Si_3_N_4_ to PETG resulted in an increase in the intensity of the phenyl ring bond at 631 cm^−1^ and a gradual increase in the intensity of the C–O–C bonds at 1616 cm^−1^. A significant decrease in intensity was also observed in the Raman peaks at 2955 cm^−1^ and 3081 cm^−1^, corresponding to the C-H2 and C-H bond stretching modes, respectively. A somewhat inconsistent behavior was observed in the 1716–1741 cm^−1^ spectral range, where C=O bonds were present. In low-concentration Si_3_N_4_ composites with compositions bearing 2 wt.%, 4 wt.%, and 6 wt.% Si_3_N_4_, respectively, an intensity decrease was found. In contrast, for the 8 wt.% and 10 wt.% Si_3_N_4_ composites, the intensity increased. This information is presented in Table 2.

### 3.2. Thermogravimetric and Differential Scanning Calorimetry Analysis

**Figure 5:**
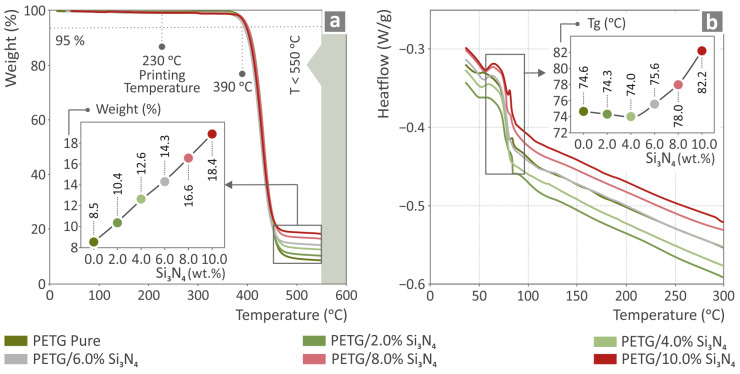
Pure PETG and PETG/x wt.% Si_3_N_4_ (x = 2.0, 4.0, 6.0, 8.0, and 10.0 wt.%) analyzed (**a**) thermogravimetrically and through (**b**) differential scanning calorimetry.

Figure 5a shows the weights of the temperature curves of PETG/2.0% Si_3_N_4_, PETG/4.0% Si_3_N_4_, PETG/6.0% Si_3_N_4_, PETG/8.0% Si_3_N_4_, PETG/10.0% Si_3_N_4_, and pure PETG as well as an inset graph of their weight loss values depending on the filler percentage. Figure 5b shows the heat flow as temperature curves for the PETG/x wt.% Si_3_N_4_ with *x* = 2.0, 4.0, 6.0, 8.0, and 10.0 wt.%, respectively, and pure PETG, along with the glass transition (Tg) temperatures for each filler percentage included in the inserted graph.

### 3.3. Viscosity and MFR Analysis

**Figure 6:**
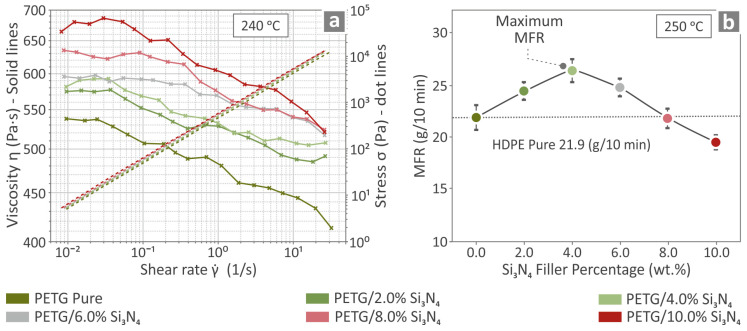
Pure PETG and PETG/x wt.% Si_3_N_4_ (*x* = 2.0, 4.0, 6.0, 8.0, and 10.0 wt.%) analyzed for (**a**) their viscosity properties at 240 °C and (**b**) their rates of melting flow at 250 °C.

The solid lines in Figure 6a represent the viscosity values of all the PETG/x wt.% Si_3_N_4_ composites and pure PETG, whereas the dotted lines indicate their stress values at 240 °C. It can be observed that as the filler percentage increased, the viscosity increased, whereas the stress was not significantly influenced. Moreover, the viscosity decreased as the stress increased. Figure 6b shows the MFR values with respect to the Si_3_N_4_ filler percentage for the PETG/x wt.% Si_3_N_4_, *x* = 2.0, 4.0, 6.0, 8.0, and 10.0 wt.% composites and pure PETG at 250 °C, at which point it is evident that PETG/4.0% Si_3_N_4_ has the highest value.

### 3.4. Monitoring of the Filament

**Figure 7:**
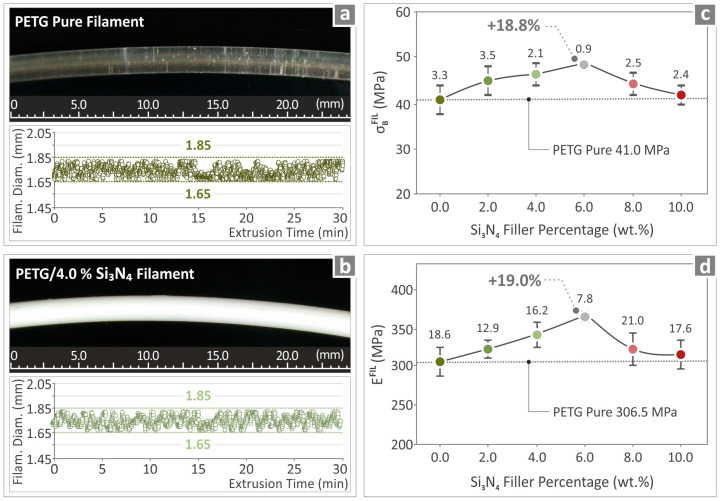
(**a**) Image of pure PETG filament and recorded measurements of its diameter, (**b**) image of PETG/4.0 wt.% Si_3_N_4_ filament and the recorded measurements of its diameter, and (**c**) tensile strength and (**d**) tensile modulus of elasticity of the filaments for all the employed filler percentages.

The extrusions of the pure PETG and PETG/4.0 wt.% Si_3_N_4_ filaments were monitored in order to observe the quality and diameter of the extruded material, and the results are shown in Figure 7a and Figure 7b, respectively, along with pictures of the materials. The filaments seem to have a high-quality surface, without defects, while also being able to maintain an almost steady diameter ranging between 1.65 mm and 1.85 mm. The ultimate strength and Young’s modulus results corresponding to those of the PETG/x wt.% Si_3_N_4_ with *x* = 2.0, 4.0, 6.0, 8.0, and 10.0 wt.%, respectively, and pure PETG filaments are contained in Figure 7c and Figure 7d respectively. The recorded values for PETG/6.0 wt.% Si_3_N_4_ were higher than those of the other composites with other filler percentages, revealing an 18.8% increase with respect to pure PETG in the case of ultimate strength and a 19.0% increase with respect to pure PETG in the case of Young’s modulus.

### 3.5. Mechanical Tests

**Figure 8:**
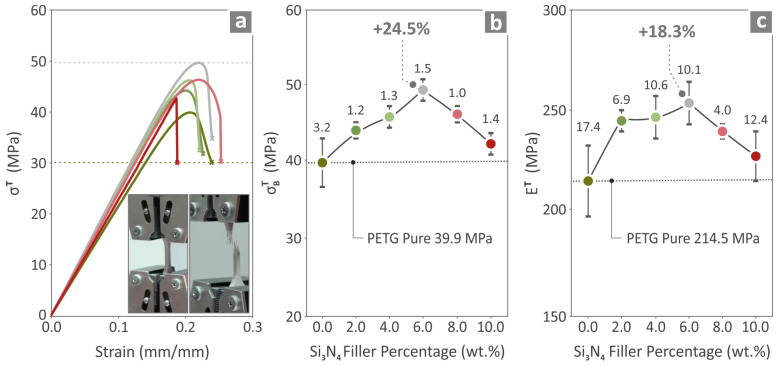
Results for pure PETG and PETG/x wt.% Si_3_N_4_ (*x* = 2.0, 4.0, 6.0, 8.0, and 10.0 wt.%) specimens about (**a**) tensile stress-to-strain graphs, (**b**) ultimate strength, and (**c**) Young’s modulus.

The prepared composites, namely, PETG/2.0 wt.% Si_3_N_4_, PETG/4.0 wt.% Si_3_N_4_, PETG/6.0 wt.% Si_3_N_4_, PETG/8.0 wt.% Si_3_N_4_, PETG/10.0 wt.% Si_3_N_4_, and pure PETG, were all tested for their tensile mechanical responses, and the results are presented in Figure 8. In Figure 8a, the tensile stress curves are shown for all the prepared composites, and in Figure 8b,c, the ultimate strength and Young’s modulus values are presented. The PETG/6.0 wt.% Si_3_N_4_ composite exhibited the highest values, with a 24.5% ultimate strength and an 18.3% Young’s modulus, higher than those of pure PETG. 

**Figure 9:**
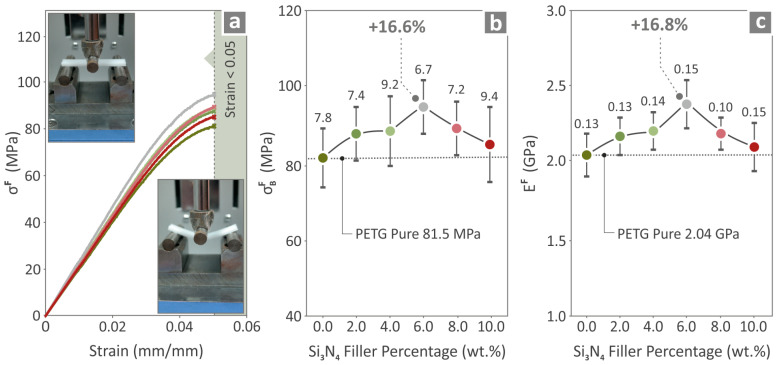
Results for pure PETG and PETG/x wt.% Si_3_N_4_ (x = 2.0, 4.0, 6.0, 8.0, and 10.0 wt.%) specimens about (**a**) flexural stress-to-strain graphs, (**b**) bending strength, and (**c**) bending modulus.

All the prepared composites, namely, PETG/2.0 wt.% Si_3_N_4_, PETG/4.0 wt.% Si_3_N_4_, PETG/6.0 wt.% Si_3_N_4_, PETG/8.0 wt.% Si_3_N_4_, PETG/10.0 wt.% Si_3_N_4_, and pure PETG were also tested for their flexural mechanical responses, and the results are shown in Figure 9. Figure 9a shows the flexural stress curves, while Figure 9b and Figure 9c present the bending strength and bending modulus values, respectively. PETG/6.0 wt.% Si_3_N_4_ showed the highest values, with a 16.6% bending strength and a 16.8% bending modulus, constituting higher values than those of neat PETG.

**Figure 10:**
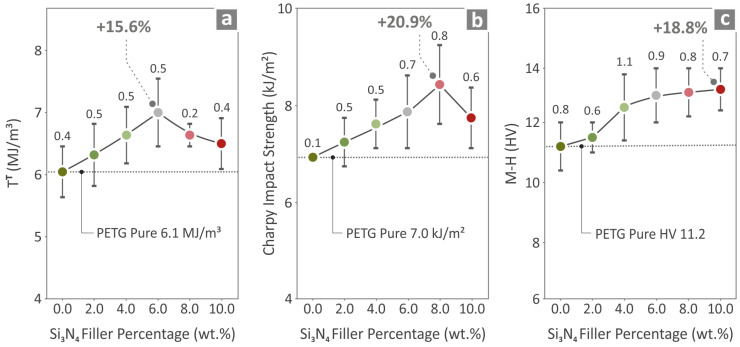
Pure PETG and PETG/x wt.% Si_3_N_4_ (x = 2.0, 4.0, 6.0, 8.0, and 10.0 wt.%) specimens’ values derived from the (**a**) tensile toughness, (**b**) Charpy impact strength, and (**c**) M-H tests.

The results for the pure PETG, PETG/2.0 wt.% Si_3_N_4_, PETG/4.0 wt.% Si_3_N_4_, PETG/6.0 wt.% Si_3_N_4_, PETG/8.0 wt.% Si_3_N_4_, and PETG/10.0 wt.% Si_3_N_4_ specimens are shown in Figure 10. Information regarding tensile toughness, Charpy impact strength, and M–H is shown in Figure 10a, Figure 10b, and Figure 10c, respectively. In relation to the pure PETG results, the composites with the highest values were PETG/6.0 wt.% Si_3_N_4_, exhibiting a 15.6% increase for tensile toughness; PETG/8.0 wt.% Si_3_N_4_, exhibiting an increase of 20.9% with respect to the Charpy impact strength; and the PETG/10.0 wt.% Si_3_N_4,_ exhibiting an increase of 18.8% as to the M-H.

### 3.6. Electrical/Dielectric Characterization

**Figure 11:**
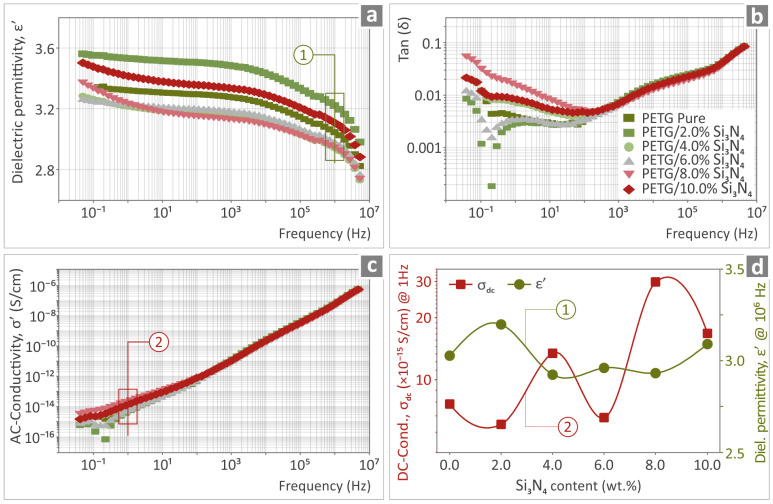
Frequency dependence of (**a**) the real part of dielectric permittivity (ε′), (**b**) the dissipation factor tan(δ), and (**c**) the real part of ac conductivity (σ′) of pure PETG and PETG/x wt.% Si_3_N_4_ composites at filler proportions ranging from 2 to 10 wt.%. (**d**) DC conductivity (σ_dc_) measured at 1 Hz and dielectric permittivity, $\varepsilon_{\infty }$ (measured at 1 MHz), as a function of Si_3_N_4_ content.

The real part of the dielectric permittivity (ε′), the dissipation factor tan(δ), and the real part of ac conductivity (σ′) as a function of the frequency of the PETG/Si_3_N_4_ nanocomposites with different filler content (0–10 wt.% Si_3_N_4_) are shown in Figure 11a–c. The dielectric permittivity of pure PETG showed no considerable frequency dispersion over the measured frequency range; that is, ε’ increases gradually from a value of 2.8 at 4 MHz to 3.3 at low frequencies, in accordance with reported values in the literature [77]. The corresponding dissipation factor exhibits values lower than 0.04 over almost the entire measured frequency range. A broad relaxation peak in the tan(δ) spectra was observed at high frequencies, located at approximately 10 kHz. This high-*f* feature remained unaffected by the addition of Si_3_N_4_ nanoparticles and can be attributed to the relaxation polarization of the polymer matrix [78]. Furthermore, an additional feature developed at low frequencies of approximately 2 Hz, which increased in intensity with an increasing filler content. This low-*f* feature can be attributed to the interfacial polarization caused by the interaction between the polymer matrix and conductive filler particles.

When the Si_3_N_4_ content was increased to 10 wt.%, we did not observe any significant spectral changes in either the dielectric permittivity or the ac-conductivity representation (Figure 11a,c). The permittivity values at high frequencies ($\varepsilon_{\infty }$) exhibited insignificant changes, varying from 2.8. to 3.4, as shown in Figure 11d. The AC conductivity spectra of the pure PETG and PETG/x wt.%Si_3_N_4_ nanocomposites show the characteristic behavior of insulating materials such as polymers; that is, ac-conductivity scales with ω at high frequencies. In the low-frequency range, a DC plateau began to develop, corresponding to the DC conductivity values of the composites varying from 5 × 10^−16^ S/cm to 6 × 10^−15^ S/cm. The variation in conductivity values measured at 1 Hz (dc-conductivity) with an increasing filler content is shown separately in Figure 11d. The above observations suggest that the overall electrical/dielectric behaviors of the PETG/x wt.% Si_3_N_4_ composites are mainly determined by the properties of the polymer matrix.

### 3.7. Analysis of Specimens through Micro Computed Tomography (μ-CT) Scanning

**Figure 12:**
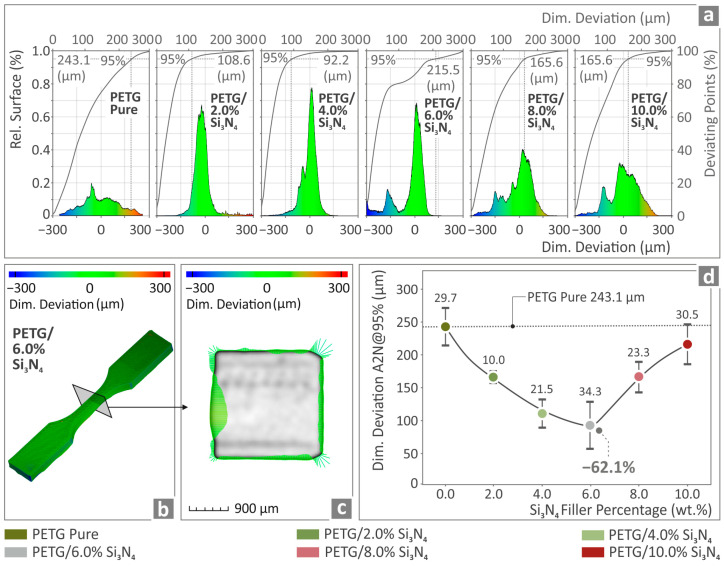
(**a**) Pure PETG and PETG/*x* wt.% Si_3_N_4_ (*x* = 2.0, 4.0, 6.0, 8.0, and 10.0 wt.%) results as to the curves corresponding to the dimensional deviation of the fabricated specimens. (**b**,**c**) PETG/6.0 wt.% Si_3_N_4_ tensile stress test results regarding the specimen’s structural deviation and (**d**) dimensional deviation A2N at 95% regarding pure PETG and PETG/*x* wt.% Si_3_N_4_ (*x* = 2.0, 4.0, 6.0, 8.0, and 10.0 wt.%).

In Figure 12a, the dimensional deviation graphs of pure PETG, PETG/2.0 wt.% Si_3_N_4_, PETG/4.0 wt.% Si_3_N_4_, PETG/6.0 wt.% Si_3_N_4_, PETG/8.0 wt.% Si_3_N_4_, and PETG/10.0 wt.% Si_3_N_4_ are presented. Figure 12b,c depict color-coded tensile stress test results for PETG/2.0 wt.% Si_3_N_4_ regarding its dimensional deviation. Figure 12d shows A2N at a 95% dimensional deviation for PETG/2.0 wt.% Si_3_N_4_, PETG/4.0 wt.% Si_3_N_4_, PETG/6.0 wt.% Si_3_N_4_, PETG/8.0 wt.% Si_3_N_4_, and PETG/10.0 wt.% Si_3_N_4_ and pure PETG, among which the value of PETG/6.0 wt.% Si_3_N_4_ was found to be 62.1% below the one corresponding to pure PETG.

**Figure 13:**
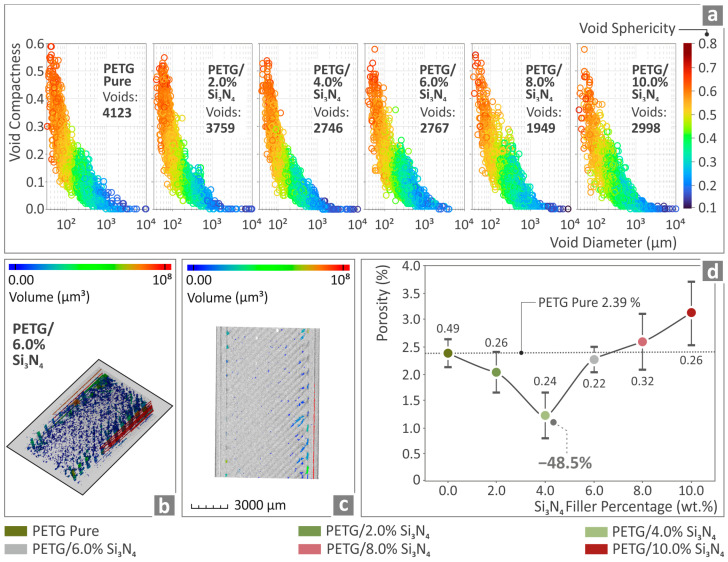
(**a**) Pure PETG and PETG/*x* wt.% Si_3_N_4_ (*x* = 2.0, 4.0, 6.0, 8.0, and 10.0 wt.%) results regarding void compactness sphericity and diameter of the fabricated specimens. (**b**,**c**) PETG/6.0 wt.% Si_3_N_4_ sample porosity in color-coding mapping and (**d**) porosity percentage regarding pure PETG and PETG/*x* wt.% Si_3_N_4_ (*x* = 2.0, 4.0, 6.0, 8.0, and 10.0 wt.%).

Relevant information regarding the void compactness, sphericity, and diameter of such characteristics for the various specimens is shown in Figure 13a for the pure PETG and all PETG/*x* wt.% Si_3_N_4_ composites. The results for the PETG/6.0 wt.% Si_3_N_4_ composite are illustrated in Figure 13b and Figure 13c, respectively, in which they are color-coded regarding voids, while the porosity percentages of pure PETG and the PETG/*x* wt.% Si_3_N_4_ with *x* = 2.0, 4.0, 6.0, 8.0, and 10.0 wt.% composites are presented in Figure 13d. It can be observed that the *x* = 4.0 wt.% Si_3_N_4_ composite presented the most promising results, being 48.5% lower than the value corresponding to pure PETG.

### 3.8. Analysis of Specimens through Scanning Electron Microscopy

**Figure 14:**
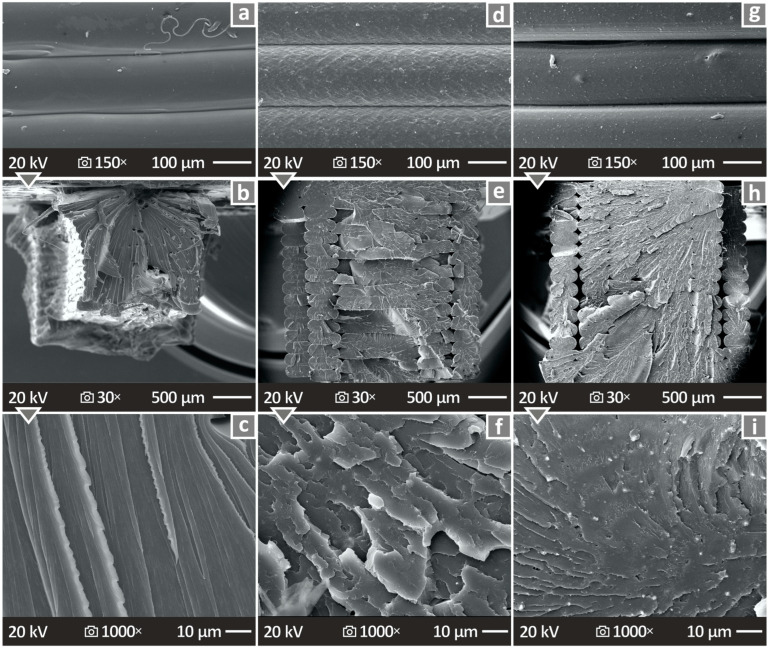
Depiction of the surfaces of the pure PETG, PETG/4.0 wt.% Si_3_N_4_, and PETG/8.0 wt.% Si_3_N_4_ samples through SEM, namely, (**a**,**d**,**g**) their side surfaces magnified 150×, (**b**,**e**,**h**) their fracture surfaces at 30× magnification, and (**c**,**f**,**i**) their fracture surfaces at 1000× magnification.

The mechanically tested specimens were subjected to SEM analysis, and relevant images of their sides and fracture surfaces are shown in Figure 14. Figure 14a,d,g depict the side surfaces of the pure PETG, PETG/4.0 wt.% Si_3_N_4_, and PETG/8.0 wt.% Si_3_N_4_, respectively, magnified 150×, indicating good layering of the material without defects. Figure 14b,e,h show the fracture surfaces of the pure PETG, PETG/4.0 wt.% Si_3_N_4_, and PETG/8.0 wt.% Si_3_N_4_ specimens, respectively, magnified 30×, presenting a solid layering with some voids. The same samples are presented in Figure 14c,f,i, where the fractured surfaces are magnified 1000×, and their high quality can be observed and confirmed, as no defects appear to be present.

**Figure 15:**
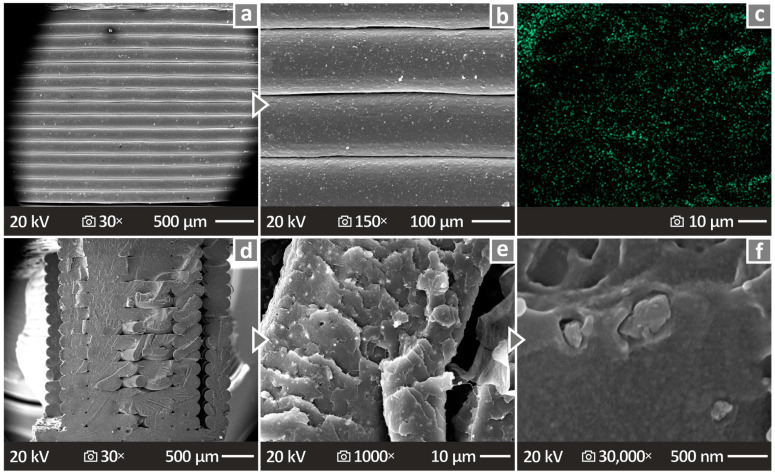
(**a**,**b**) Depiction of the PETG/10.0 wt.% Si_3_N_4_ sample’s side surface via SEM, magnified at 30× and 150×, respectively; (**c**) EDS mapping picture of a chosen sample; (**d**–**f**) depiction of the PETG/10.0 wt.% Si_3_N_4_ sample’s fracture surface via SEM, magnified at 30×, 1000×, and 30,000×, respectively.

Side surface images of a PETG/10.0 wt.% Si_3_N_4_ specimen can be observed in Figure 15a,b, magnified at 30× and at 150× respectively, indicating great layer fusion. Figure 15c shows an EDS mapping image, while Figure 15d–f present the fracture surface images of PETG/10.0 wt.% Si_3_N_4_ at 30×, 1000×, and 30,000× magnifications, respectively. The revealed structure was determined to be of high quality because the defects and voids were minimal, except in the case of Figure 15f, where some areas showed uneven pieces of material.

## 4. Discussion

The authors used a typical thermomechanical process for filament preparation, constituting the standard in the industry for both pure polymeric materials and composites. They chose to use this approach as it can easily be scaled up and industrialized. The drying process was used to remove any remaining moisture from the atmosphere first from the raw materials and the filament afterward. This is also a typical method and entails removing moisture from the atmosphere. The production of the filament did not include any solutions or other liquids that would need to be removed using filtration processes afterward. Additionally, a thorough process was followed to ensure the quality of the produced filament, as presented in this manuscript. This included inspection of random parts of the filament under a microscope to locate possible defects and qualitatively evaluate its surface roughness, measuring its diameter, and finally conducting tensile tests to assess its strength. In the mechanical tests, no high deviations were found between the samples, indicating a similar composition and structure between the filament samples. The tensile test results are comparable to those of the respective 3D printed samples, although such a comparison cannot be considered reliable. The filaments were not tested according to a standard, as the authors are not aware of such a standard for filament tensile testing.

**Figure 16:**
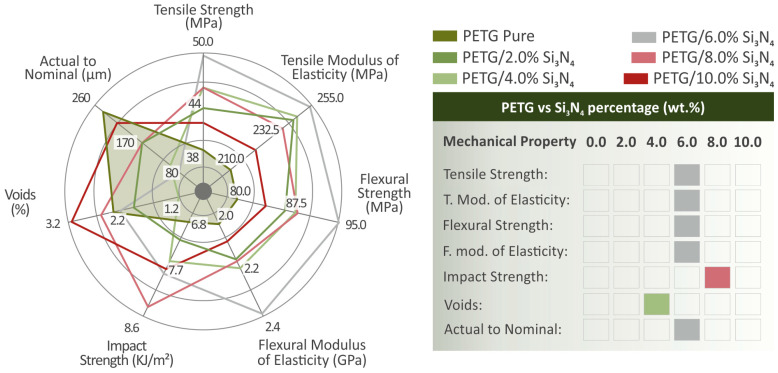
(**On the left side of the figure**) The examined mechanical properties’ values are summarized in a spider graph regarding pure PETG and PETG/*x* wt.% Si_3_N_4_ (*x* = 2.0, 4.0, 6.0, 8.0, and 10.0 wt.%). (**On the right side of the figure**) Data board highlighting the composites wherein each mechanical property presented the greatest result.

The findings on the mechanical properties revealed reinforcement in relation to the behavior of pure PETG, especially for a 6.0 wt.% Si_3_N_4_ filler percentage, which presented the most enhanced properties among all the various composites synthesized. The dielectric properties were examined, and the results suggested that the polymer matrix mainly affected overall electrical behavior. The structures of the specimens were observed using an electron microscope (SEM) at various magnifications. These SEM images show the very good layering and inner structures of the various specimens, which were not negatively affected by the addition of different filler percentages of Si_3_N_4_. The dimensional deviation results were significant in the case of the 6.0 wt.% Si_3_N_4_ composite, while in the case of 4.0 wt.% Si_3_N_4_, the porosity percentage was the lowest among all the different composites synthesized. The EDS analysis showed a relatively good dispersion of the additive, which verified the excellent results for sample structure derived from the rest of the tests. On the left side of Figure 16, the spider graph shows all the mechanical properties of the PETG/*x* wt.% Si_3_N_4_ and pure PETG samples, whereas on the right side of Figure 16, the mechanical properties are matched with the composites in which their greatest values were detected. This enhancement in the mechanical properties can be attributed to the interactions between the nanoparticles and the matrix as well as the restriction of polymer chain mobility resulting from the nanoparticles occupying the spaces between them [79,80,81].

Regarding the physical properties, rheology was affected, as discussed. This has an effect on the 3D-printed structure, which provides information for the optimization of the 3D-printing settings for each composite to maximize mechanical performance. For comparison purposes, this optimization was not performed in this study. Regarding the brittleness of the samples, no large differences were found between the unfilled PETG and the nanocomposites. The inspection of the fracture surfaces showed minimum deformation in both the pure PETG and the nanocomposites. At the same time, the 3D-printed samples failed at similar strain values for tensile strength. Only the higher-loaded samples failed at lower strain values, revealing inferior mechanical strength. This can be attributed to the saturation of the filler in the matrix, which negatively affects mechanical performance [79,82].

It is important to highlight that the scanning electron microscopy (SEM) images displayed finely crafted surfaces and a uniformly distributed layering of the materials, even after the addition of fillers. A few voids were detected, and any defects observed were minimal in size. The incorporation of Si_3_N_4_ nanoparticles influenced the rheological behavior of the PETG polymer, resulting in an overall increase in viscosity. The MFR increased to 4 wt.% Si_3_N_4_ content in the composites and drastically reduced at higher loadings. Despite these changes, there were no significant disparities in the quality of the 3D-printed parts. The fusion of layers, as evidenced by the images of the lateral surfaces, appeared to remain intact, even for the higher-loaded nanocomposites, indicating a consistent layer thickness.

Inspection of the internal structure via micro-computed tomography (μ-CT) corroborated these findings. The addition of Si_3_N_4_ enhanced dimensional accuracy and reduced the number of voids in the internal structure. Notably, nanocomposites with superior mechanical performance exhibited fewer voids and better dimensional accuracy, implying a correlation between printing quality and mechanical strength. This is in agreement with the literature, which indicates that increased porosity negatively affects the mechanical performance of 3D-printed parts [83,84]. In contrast, the addition of 4 wt.% composite showed the minimum porosity among the samples tested, showing that other factors affected the mechanical strength of the samples besides porosity. The 6 wt.% composite showed the best dimensional accuracy among the samples assessed, which is an indication of good 3D-printing quality as well, showing that good 3D-printing quality positively affects the mechanical performance of the 3D-printed parts.

The thermal property analysis indicated that there was a negligible impact of Si_3_N_4_ addition on the PETG response to high temperatures, confirming the safety of the temperature levels used and the absence of thermal degradation. Tg increased only slightly in the composites with higher filler loadings. SEM and energy-dispersive X-ray spectroscopy (EDS) did not reveal major particle clustering in the fracture surfaces, even in the higher-loaded composites. No agglomerations were located in the nanocomposites with lower Si_3_N_4_ content. Moreover, the mechanical tests revealed that the deviation in results remained within acceptable limits, which suggests that the composition of the nanocompounds was consistent across all the examined samples. Therefore, it is safe to assume that a good dispersion of Si_3_N_4_ nanoparticles in the PETG matrix was achieved using the process followed for the preparation of the nanocomposites. The raw material mixing process and the filament extruder used, the latter of which is specially optimized for material mixing, contributed to this result. Even the highest-loaded samples exhibited slightly superior mechanical performance compared to pure PETG. A decreasing trend was found, indicating possible saturation of the filler, although the saturation threshold of the Si_3_N_4_ filler in the matrix was not precisely determined, as such information fell outside the scope of this study.

After reviewing the related literature, similar investigations were performed to examine Si_3_N_4_ as a filler and its reinforcing properties in nanocomposites. In ref. [85], Si_3_N_4_ was employed as an additive in the matrix material of polypropylene (PP) to investigate its effect on the mechanical and electrical properties of PP, as also observed in this study, in which PETG was used as the matrix material. The results indicate an increase in impact strength and a decrease in tensile strength, which is in disagreement with the tensile strength results of the study. This behavior may be attributed to the different matrix materials used in each study.

In another study [60], Si_3_N_4_ was added to two polymer matrix materials: high-density polyethylene (HDPE) and polypropylene. The 3D-printed parts of the polymer–ceramic material presented smooth surfaces without ledges or discontinuous areas, a result that was also observed in the samples in this study. Moreover, the filler content of 10 wt.% did not cause any remarkable reinforcement of the elastic modulus, and the critical strain decreased. In ref. [42], biomedical-grade PLA with various filler loadings was employed as a matrix material for Si_3_N_4_. A series of tests were conducted on the composites, indicating an increase in flexural and tensile strength, as also observed in the present work. In Table 3 below, a comparison of the effects of Si_3_N_4_ as a reinforcement agent on different polymeric matrices is presented. As shown, there are differences in the performance of Si_3_N_4_ as a reinforcement agent between the polymeric matrices, but overall, the results can be considered comparable. Any differences can be attributed to the different polymeric matrices used and the differences in the preparation process of the composites.

## 5. Conclusions

Composites consisting of PETG and Si_3_N_4_ were synthesized in appropriate mixtures and used to produce filaments suitable for the fabrication of various specimens using MEX 3D-P. Specifically, composites with 2.0, 4.0, 6.0, 8.0, and 10.0 wt.% Si_3_N_4_ filler quantities were shaped into mixtures, filaments, and finally specimens. Samples originating from the raw materials, the filaments, and the final 3D-P specimens underwent specific tests, with the aim of investigating their properties and performance under specific conditions. SEM was used to investigate the structures of all the specimens. An EDS analysis was conducted to examine the chemical compositions of the composites and raw Si_3_N_4_ to reveal the expected chemical elements, thus confirming the quality and homogeneity of the prepared materials. The electrical and dielectric properties pertaining to BDS were investigated. Raman spectroscopy, TGA, and differential scanning calorimetry (DSC) were performed. The viscosities and MFR were also investigated. The tensile performance of the filaments was tested. A similar experiment was performed on the specimens to determine their flexural properties, Charpy impact strength, and microhardness. The dimensional deviation and void percentages of the various samples were also examined.

The derived results revealed a remarkable enhancement in PETG/6.0 wt.% Si_3_N_4_ performance for the majority of the properties. Additional work could include observing the influence of different 3D-P settings on the performance of the samples. Thus, elucidating the influence of 3D-P settings on the overall performance of the final products can have a tremendous impact on the creation of various parts used in anti-ballistic protection [86], including various armor parts used in personal protective gear, such as helmets, breastplates, or joint protective gear for kneepads and elbows. All of the above can have a significant positive impact on the defense and security industry, revolutionizing applications and reducing manufacturing costs significantly.

## Figures and Tables

**Table 1:** Significant Raman peaks and their related assignments from pure PETG.

Wavenumber (cm^−1^)	Intensity	Raman Peak Assignment
631	Strong	phenyl ring vibration [68,69]
703	Medium	C-H out-of-plane bending [68]
772	Small	O-C(O)-O stretching [69]
793	Medium	
855	Strong	γ(C–OH)_ring_ [70,71]
1115	Strong	Skeletal vibrations, C–O–C bonds [72]
1172	Strong	Skeletal vibrations, C–O–C bonds [68,72]
1283	Strong	Skeletal vibrations, C–O–C bonds [68,72]
1369	Small	C–C–H, C–O–H, and O–C–H [72]
1409	Medium	C-H_3_ deformation [73]
1440–1464	Medium	C-H_3_ deformation [68,73]; C-H_2_ deformation [72]; C-H_3_ symmetric bending [69,73,74]
1613	Very Strong	Phenyl ring stretch [69]
1724	Very Strong	C=O bond [75]
2857	Medium	C-H_2_ symmetric stretching [72]
2890	Medium	CH_2_ symmetric stretching [72,76]
2955	Strong	CH_2_ asymmetric stretching [72]
3081	Strong	C-H stretching [73]

**Table 2:** Significant Raman peak differences between the PETG/Si_3_N_4_ samples and PETG/pure.

631	Peak rise	Small increase in phenyl ring bond
1616	Gradual increase	Significant increase in skeletal vibrations or C–O–C bonds
1716–1741	Inconsistent behavior	C=O bond [75]
2955	Peak drop	Significant decrease
3081	Peak drop	Significant decrease

**Table 3:** Comparison with the literature regarding the findings on the effect of using Si_3_N_4_ as an additive in polymeric matrices on mechanical properties.

	Current Study	[42] (PLA Matrix)	[55] (ABS Matrix)	[59] (PP Matrix)	[85] (PP Matrix)
Impact strength increase	20.9%	30.2%	Decrease	11.1%	25%
Tensile strength increase	24.5%	40.4%	25.6%	16%	Decrease
Flexural strength increase	16.6%	33.2%	30.3%	15.7%	-
microhardness	18.8%	20.9%	34.9%	33.6%	-

## Data Availability

The raw/processed data required to reproduce these findings cannot be shared because of technical or time limitations.
